# Facts and Principles as to Hospital Reform

**Published:** 1897-11-20

**Authors:** 


### FACTS AND PRINCIPLES AS TO HOSPITAL REFORM.

In view of the importance of the question of hospital reform, which has now for some time back been so anxiously discussed both by the public and the medical profession, as well as by those who are more immediately concerned in hospital management, we think it well to state certain facts which must be borne in mind, and to formulate certain general principles which, in our opinion, must be acted upon if any permanent good is to be done.

The first fact, then, is that hospitals are charities, and that the very reason for their existence is that those who receive their benefits receive something which they cannot buy.

The next fact is a corollary of the first, and is this, that those who can afford to pay for the sort of help which is given by the hospitals are entirely unfit objects for their charity, and that no payment should allow such people to buy hospital treatment.

The next fact is, that those who require medical attendance, and yet are unable to pay for it, are fit objects for hospital treatment. If this were all, and if the population could be neatly divided into the fit and the unfit, the problem would be solved at once; but this is not the case. The real difficulty comes in with the last fact we have to mention, viz., that between those who can pay for all and those who can pay for none is a great middle group —people who can pay for ordinary attendance but not for extraordinary—for it is from sympathy with such people that hospitals have been led to slacken their rules and accept a class of patients who, as the general practitioner says, are "quite able to pay."

In reality these people, although often fairly well off, may be truly objects of charity, and no great harm is done so long as they are suffering from ailments which do in fact require hospital treatment. Unfortunately they have been followed by a horde of people who do not frequent the hospitals so much for the sake of hospital treatment as for the sake of cheap treatment; and the true and the useful and the good hospital reform—the only permanent remedy for hospital abuse—is, in our opinion, some workable means for keeping out those who, although they can pay, seek cheap attendance, and letting in those who, although they can pay something, cannot obtain the treatment they require except either in a hospital or at fees quite beyond their means. This being so, it is hopeless to trust to honour, or to common honesty, or to any of the virtues to cure abuse, for the very abuse we want to cure is the intrusion of people who are callous to all these things. A sieve of some sort must be found, and the first principle we would lay down is that the case of every applicant for hospital treatment should be investigated by a specially appointed inquiry officer, who should weed out and put absolutely on one side those able to pay for what they require. What, then, about those who are able to pay for ordinary attendance, but come on the plea that what they require is something different, something for which they cannot afford to pay? Here comes in the second principle: Make them subscribe, not according to or for the treatment required, but according to their means. This is not, and must never be considered a payment for what is received; to these, as to the poorest, the benefit given is a charity; but this forced contribution would at least have the effect of keeping away the mean seekers for cheap physic. The general practitioner is naturally angry, and the subscriber is naturally disgusted, when he sees patients who are quite able to pay their half-crowns making use of the hospital for every little ailment. It is a great abuse. But neither the practitioner nor any one else objects to that same patient using the hospital for some special malady for which he could not otherwise get properly treated except by payment of high fees, which he clearly could not afford. The subscription of such a sum as he could afford would do this man no harm, but it would keep the cheap physic-seeker out; and it would prevent the hospitals competing with and underselling private practitioners. The subscription by those able to pay is to be looked at as a means of keeping the unfit out, not of letting the unfit in. If no one who is able to pay ordinary medical fees is admitted to hospital unless he subscribed an equivalent amount, none will go to hospital except those who seek that special help which the hospitals alone can give, and which they cannot afford to buy; and to such people no one objects.

How this money is to be collected is another affair. A simple method of doing it by means of the Prince of Wales's Fund stamps has been suggested, but that is a detail. What we think is important is the acceptance of the two great principles, that applicants must pass the inquiry officer, and next, that those who are not indigent must be made to contribute *according to their means.* The contribution demanded from those who are fairly well off might come to far more than they would pay for ordinary attendance outside; and when it at all approached the private fee of the physician or surgeon, we may be sure the patients would seek a private consulting-room.

